# 人体菌群与肺癌的治疗相关性

**DOI:** 10.3779/j.issn.1009-3419.2019.07.09

**Published:** 2019-07-20

**Authors:** 卉洁 周, 娇娇 锁, 江 朱

**Affiliations:** 610041 成都，四川大学华西医院胸部肿瘤科 Department of Toracic Oncology, West China Hospital, Sichuan University, Chengdu 610041, China

**Keywords:** 肺肿瘤, 人体菌群, 化疗, 放疗, 基因治疗, 免疫治疗, Lung neoplasms, Human microbiome, Chemotherapy, Radiotherapy, Gene therapy, Immunotherapy

## Abstract

人体菌群与人类健康状态密切相关，如人体菌群的失调可能导致糖尿病、胃肠道疾病、肥胖等疾病的发生。人体内微生物与约20%的恶性肿瘤有关，肺癌（lung cancer, LC）是目前最为常见的恶性肿瘤之一，我国男性LC发病率及死亡率高居所有恶性肿瘤之首。近来研究表明，人体菌群可能通过代谢、炎症或免疫等途径影响着LC的发生，同时影响LC对放化疗、基因治疗、免疫治疗等治疗方法的疗效，如免疫治疗，是用于治疗LC的一种极有前景的手段，但不同患者从中获益不一，包含以肺癌细胞株的实验表明肠道微生物群可通过与宿主免疫系统的相互作用调节对免疫治疗的反应。但针对肺癌患者，肠道菌群是否仍能对免疫治疗进行调节仍存在争议。本文就人体菌群与LC的治疗相关性的近来研究进展进行综述。

## Abstract (en)

Te human microbiome is closely related to human health status. Disruption of the symbiotic balance of the human microbiome is commonly found in systematic diseases such as diabetes, obesity, and chronic gastric diseases. Te human microbiome confers benefts or disease susceptibility to the human body through multiple pathways, associated with approximately 20% of malignancies. Te incidence and mortality of lung cancer (LC) in men in China are the highest among all malignancies, which is a serious threat to human health. Emerging evidence has suggested that the human microbiota may be closely related to lung cancer at multiple levels, *e.g*., by affecting metabolic, inflammatory, or immune pathways. At the same time, the human microbiota affects the efcacy of lung cancer on chemoradiotherapy, gene therapy, immunotherapy and other treatments. Immunotherapy is a promising method for the treatment of malignancies such as lung cancer, but the efcacy of immune checkpoint inhibitors in patients is heterogeneous. Preclinical studies based on lung cancer cell lines suggest that the intestinal microbiota can modulate responses to anti-PD-1 therapy through interactions with the host immune system. But for lung cancer patients, whether the intestinal flora can still regulate immunotherapy remains controversial. In this mini-review, we summarize current research fndings describing therapeutic relevance of human microbiota and lung cancer. A beter knowledge of the interplay between the human microbiome and lung cancer may promote the development of innovative strategies for prevention and personalized treatment in lung cancer.

## 1 人体菌群简介

人体菌群，被称为“人类的另一个基因组”^[1-4]^，主要分布在胃肠道、呼吸道、泌尿生殖道及皮肤表面，影响着宿主的营养状态、物质的新陈代谢及生理和免疫功能^[5, 6]^。肠道是容纳人体菌群数量最多的部位，约1.5 kg的细菌定植于此。肠道细菌及其代谢产物通过肝肠循环或受损的肠道粘膜进入血液循环，其中益生菌参与人体的新陈代谢、营养物质合成及炎症反应；而致病菌通过影响神经、免疫系统，促进肥胖、糖尿病、癌症等疾病的发生^[7, 8]^。然而与肠道菌群相比，呼吸道菌群的研究热度明显减少，这与“下呼吸道为无菌环境”的历史观点不无关系。近年来研究发现下呼吸道仍然存在着共生菌群，其组成与上呼吸道相似，菌群数量从上呼吸道至下呼吸道呈阶梯式减少，据此推测上呼吸道的菌群可能通过呼吸到达下呼吸道^[9]^。目前研究证实呼吸道的菌群失调与囊性纤维化（cystic fibrosis, CF）、慢性阻塞性肺疾病（chronic obstructive pulmonary disease, COPD）、哮喘等疾病密切相关^[10-12]^。口腔菌群的数量仅次于肠道菌群，是人体寄生菌群第二多的部位^[13]^，口腔菌群与某些疾病如龋齿、牙周炎、口腔肿瘤等疾病的发生密切相关，如链球菌、放线菌和乳酸杆菌等糖酵解菌，通过将碳水化合物降解为有机酸，引发龋齿^[14]^；普氏菌和卟啉单胞菌等氨基酸降解菌，通过将蛋白质和多肽降解成短链脂肪酸，含氨、硫化合物以及吲哚、粪臭素等，导致牙周炎和口腔恶臭^[15]^。口腔中的核梭杆菌，念珠菌等更是与口腔恶性肿瘤不无关系^[16]^。生殖道菌群是一个以乳酸杆菌为主动态平衡的微生态系统，通过将阴道鳞状上皮细胞内的糖原分解成乳酸，使阴道局部形成弱酸性环境来抑制其他寄生菌的过度生长，维持阴道微生态环境的平衡。

## 2 菌群与肺癌（lung cancer, LC）的发生

流行病学调查显示，全球恶性肿瘤中发病率、死亡率最高的均为LC。但与哮喘、COPD、CF等肺部疾病相比，目前研究尚不能完全阐明人体菌群在LC发生中的作用与机制^[17-20]^。

有多项研究发现LC的发生与人体菌群存在一定相关性：人体呼吸道中嗜木聚糖真杆菌、挑剔真杆菌及梭菌属的增多可能与小细胞肺癌（small cell lung cancer, SCLC）的发生率呈正相关；而普氏菌、瘤胃假丁酸弧菌的数量却与SCLC的发生可能存在负相关关系^[21]^。在无吸烟史的人群中，将LC患者与非LC患者的痰标本进行对比，从LC患者痰标本中分离出更多的*Granulicatella*、*Abiotrophia*和*Streptococcus*^[22]^。LC组织中的*Modestobacter*相较于邻近正常组织更多；而丙酸杆菌、肠杆菌科含量较少^[23]^。而抗生素如青霉素、头孢菌素的使用，改变了菌群数量及构成，与此同时，LC患病风险随之增加^[24]^。

以上研究，支持人体菌群的变化可能会推动LC的发生这一观点，究其机制，有研究者^[25, 26]^认为：肺部菌群可能以肺部炎症为触发点，进而促进LC的发生。肺部微生物群的结构和数量变化可以加速COPD炎症状态的进展；而大量证据表明COPD与LC发生之间存在关联^[27, 28]^。但炎症产生的原因以及其特异性免疫介质是什么？近来一项研究表明：LC的发展与局部的菌群失调和炎症有关。肺部共生菌群通过激活肺部驻留的gd T细胞引起与肺腺癌相关的炎症。在消除共生菌群后，肺腺癌的发生率显著降低。共生菌群刺激骨髓细胞产生Myd88依赖性IL-1b和IL-23，诱导Vg6+Vd1+gd T细胞的增殖和活化，产生IL-17和其他效应分子，促进炎症发生，导致肿瘤细胞增殖^[29]^。

而与此观点相反，一项小样本研究对比了肺部肿瘤组织与邻近正常肺组织，结果并未发现肿瘤组织中存在特异性的菌群改变^[30]^。是否LC组织中存在菌群的变化，以及这些变化是否促进了癌症的发生，仍需进一步探索。

## 3 菌群与LC的治疗

### 3.1 菌群与化疗

化疗是LC患者的主要的治疗手段之一。而化疗，这种细胞毒性治疗，会造成LC患者菌群的显著变化：导致肠道益生菌减少，致病菌增加。以铂类为基础的两药联合方案是晚期LC患者的标准一线化疗方案，有研究通过对30例接受含铂方案化疗的LC患者治疗前后的粪便菌群进行分析发现：化疗后肠道内的益生菌如双歧杆菌、乳酸杆菌、柔嫩梭菌属、胃球菌属的数量减少，致病菌如肺炎克雷伯杆菌数量增加^[31]^。上述结果与另两项研究——SCLC经依托泊苷+顺铂（Etoposide+Cisplatin, EP）方案化疗后梭状芽胞杆菌增多^[32]^，NSCLC经含铂双药化疗后挑剔真杆菌数量增加^[33]^——结果基本一致。而这种致病菌的增加是否与化疗后免疫力下降导致感染几率增加有关尚不清楚，以及这种菌群变化对LC患者的预后是否会产生影响仍需进一步探索。

目前还发现肠道共生菌群可能参与了抗肿瘤反应。益生菌联合化疗可能具有协同作用：针对Lewis肺癌小鼠模型的研究显示，顺铂联合ABX（vancomycin, ampicillin, and neomycin, ABX）一种可以破坏宿主共生微生物群的抗生素混合物，由万古霉素、氨苄青霉素和新霉素混合制成，处理荷瘤小鼠，其肿瘤生长速度显著超过单用顺铂处理的小鼠，存活率亦显著降低；而顺铂联合乳酸杆菌处理的小鼠，肿瘤被良好控制且存活率更高^[34]^。研究者推测有益共生菌群有助于顺铂的抗肿瘤作用。对基因表达的进一步研究表明，顺铂联合ABX可以通过上调癌基因血管内皮生长因子A（vascular endothelial growth factor A, VEGFA）的表达，下调抑癌基因*BAX*和*CDKN1B*的表达来部分降低顺铂的疗效。ABX共处理小鼠的CD8^+^ T细胞中IFN-γ、GZMB和PRF1的表达降低，表明肠道菌群结构被破坏后机体抗肿瘤免疫作用也随之降低；相反，乳酸杆菌共处理小鼠显示出增强的抗肿瘤免疫反应。另有研究^[35]^发现，晚期LC患者在接受以铂类为基础的化疗后，其体内特异性识别*E. hirae*和*B. intestinihominis*的记忆性Th1细胞的数量越多，预示着更长的无进展生存期（progression-free survival, PFS）。

### 3.2 菌群与放疗

LC患者在放疗期间容易发生肺部感染。接受根治性放疗后发生感染的局部晚期NSCLC患者，其病原菌主要分布于呼吸道，以大肠埃希菌、铜绿假单胞菌、金黄色葡萄球菌、表皮葡萄球菌为主^[36]^。有研究对240例行放化疗治疗的LC患者临床资料进行分析，发现发生肺部感染的感染率为19.17%，且感染患者均为复合感染^[37, 38]^。回顾性调查分析252例住院行同步放化疗的局部晚期NSCLC患者，其中58例发生院内感染（占23.02%）^[39]^。局部晚期非小细胞LC患者接受放疗后，呼吸道菌群可能发生改变，从而导致院内感染的发生，应重点予以关注。

关于LC放疗副反应方面，有研究^[40, 41]^发现口服补充益生菌可增加双歧杆菌、乳酸杆菌、肠球菌等优势有益菌，减少大肠杆菌等潜在致病菌。有效调节放疗后肠道菌群失调，能预防、治疗放疗所致急性放射性肠炎发生。此外，目前关于菌群与放疗效果的研究还非常有限，值得进一步探索。

### 3.3 菌群与基因治疗

双歧杆菌是肠道固有的无致病性厌氧菌，能特异地聚集于乏氧的肿瘤组织：因乏氧肿瘤组织中新生血管的内皮系统尚未完全形成，故双歧杆菌能靶向性地进入到肿瘤组织中^[42, 43]^。研究^[44]^显示：将自杀基因和免疫调节因子等导入双歧杆菌，这些双歧杆菌将在包括LC在内的实体肿瘤中特异性的增殖，发挥抗肿瘤作用。例如：可溶性类fms酪氨酸激酶-1（soluble fms-like tyrosine kinase receptor, sFlt-1）是一种具有酪氨酸激酶活性的血管内皮生长因子（vascular endothelial growth factor, VEGF）抑制物，利用双歧杆菌介导的sFlt-1基因转导系统可以在基因和蛋白质水平上表达sFlt-1，能显著抑制血管内皮细胞的生长，更有效、安全地控制小鼠Lewis LC的生长^[40]^。因此，双歧杆菌可作为包括LC在内的实体瘤基因治疗的合适载体，可能在LC未来的基因治疗发展中发挥重要作用。

### 3.4 菌群与免疫治疗

免疫治疗在晚期LC治疗领域发展迅速，取得了LC一线适应证并广泛用于LC的临床治疗。临床上最重要的免疫检查点是细胞毒性T淋巴细胞相关抗原4（cytotoxic T-lymphocyte-associated protein 4, CTLA-4）和程序性细胞死亡蛋白-1（programmed death-1, PD-1）及其配体1（programmed cell death protein 1 ligand 1, PD-L1）。通过阻断免疫检查点，可以使已经对肿瘤免疫耐受的T细胞重新被激活，激活的T细胞可有效识别并攻击肿瘤细胞。然而，免疫检查点阻滞剂仅使部分晚期LC患者受益，导致疗效差异的部分原因可能是患者体内微生物的组成差异。

Routy等^[45]^的研究中观察了包括140例晚期NSCLC在内的共249例上皮性肿瘤患者，69例在第一次接受免疫检查点阻滞剂（immune checkpoint inhibitors, ICI）前后2个月内接受过抗生素（antibiotics, ATB）治疗。单独对晚期NSCLC患者进行分析，发现ATB暴露组的PFS、OS缩短，这一结果同整体分析结果一致。多变量*Cox*回归分析显示，在NSCLC及RCC患者ICI疗效预测中，抗生素暴露是独立于经典预测因子的又一疗效预测因子。另一项包含了239例晚期NSCLC患者的研究^[46]^，将48例免疫治疗前30 d内接受过抗生素治疗的患者与未接受过抗生素治疗的患者进行比较，结果同样显示，抗生素暴露人群的中位PFS与中位OS降低，ATB对于NSCLC患者OS的影响显著。

既往已有研究^[47]^表明ATB可以改变肠道微生物组的组成。进一步分析抗生素暴露与无抗生素暴露两组NSCLC患者的肠道菌群，发现*A. muciniphila*与ICI疗效显著相关。为了证实其相关性，使用ATB对Lewis肺癌小鼠进行微生物群灭菌，再口服补充*A. muciniphila*，ICI抗癌效果恢复，以荷RET黑素瘤小鼠为对象，亦得出相同结果。利用ICI治疗未获益者的粪便，对无特定病原体（specefic pathogen free, SPF）小鼠进行粪便菌群移植（fecal microbiota transplantation, FMT），经处理后的小鼠亦不能从ICI治疗中获益；但继续口服补充*A. muciniphila*，小鼠体内更多的CCR9^+^ T淋巴细胞、CXCR3^+^ T淋巴细胞、CD4^+^ T淋巴细胞以白细胞介素-12依赖性方式向瘤床的募集，小鼠得以从ICI治疗中获益^[45]^。

以上研究以肺癌小鼠及患者为研究对象，证实了肠道菌群对PD-1阻滞剂疗效的显著影响。而近来我国一项研究对37例接受ICI治疗的晚期NSCLC患者进行分析^[48]^，发现：治疗响应者的基线肠道微生物多样性更高，并且在治疗期间其组成更为稳定。而肠道微生物多样性更高的患者，PFS显著延长。在响应者粪便中，*Alistipes putredinis*、长双歧杆菌、普氏菌的含量更高，而非响应者的粪便中，未分类的*Ruminococcus*含量更丰富。进一步分析患者全身免疫反应，结果显示肠道微生物组多样性更高的患者，在使用纳武利尤单抗后产生了更多的记忆CD8^+^ T细胞及NK细胞亚群。该研究揭示了中国晚期NSCLC患者的肠道微生物多样性与ICI疗效之间的强相关性。

以上临床前研究及临床研究结果均显示，肠道菌群影响着LC的ICI疗效，然而有研究得出了相反的结论。

两项回顾性研究调查了抗生素暴露是否会影响nivolumab对NSCLC患者的疗效。一项研究分析了90例接受nivolumab治疗的NSCLC患者。13例患者存在抗生素暴露。发现抗生素暴露组较未暴露组的PFS显著缩短。两组生存曲线之间的差异具有统计学意义。然而在多变量分析中，抗生素暴露与ICI疗效之间并未显示出显著相关性^[49]^。另一项研究纳入了74例NSCLC患者。在接受nivolumab治疗前后3个月内，15例（20.3%）患者接受过抗生素治疗。统计发现抗生素暴露并未对免疫治疗反应率（CR或PR）及PFS造成影响。该研究认为使用抗生素导致的菌群改变对nivolumab在NSCLC患者中的疗效似乎不造成影响^[50]^。

分析造成结论差异可能的原因，在不同研究中，定义“抗生素暴露”的时间节点不一致，微生物群在ATB中断后1个月-3个月内恢复到其基线状态，而某些细菌可能需要数年才能完全恢复^[47]^。ATB的类型、给药持续时间、给药途径的不同，是否会造成抗生素暴露后不同的菌群状态，以及发生改变的菌群是否为“免疫治疗有益菌”，亦会对结果造成影响。而研究中是否排除了其他如抗酸剂，非甾体抗炎药等其他影响菌群的药物，也是影响研究结果的因素。

目前大量针对多种癌种的研究证实了肠道菌群在调节ICI疗效中的作用^[51-57]^。补充双歧杆菌能有效控制荷黑色素瘤小鼠体内肿瘤生长，增强抗PD-L1药物的抗肿瘤作用^[58]^。从PD-1治疗中获益的黑色素瘤患者肠道内微生物多样性更高，Ruminococcaceae的丰度更高^[59]^。多个不同模型的动物实验证实ICI在无菌小鼠或抗生素处理的荷瘤小鼠中几乎无效，但将含免疫治疗“有益菌”（如，*Akkermansia*、*Faecalibacterium*、*Bifidobacterium longum*、*Collinsella aerofaciens*、*Enterococcus faecium*和*B.fragilis*）的免疫治疗响应者的粪便微生物移植到无菌荷瘤小鼠体内，则ICI的疗效不同程度提高^[59-62]^。有学者进一步认为，肠道中对免疫治疗“有益菌”与“无益菌”比例或许是ICI最有效的疗效预测指标^[60, 62]^。

## 4 总结与展望

人体是复杂多样的微生物栖息地，人体菌群有助于维持宿主的整体稳态。越来越多的基础研究逐渐发现人体菌群对恶性肿瘤如LC的发生发展以及治疗、预后等有着重要影响，其临床价值尚待更多随机对照试验及真实世界研究进一步发现和验证。有望通过“设计益生菌”等手段调节肠道菌群，从而改善LC患者疗效和预后。
